# Adaptive Deep Brain Stimulation for Movement Disorders: The Long Road to Clinical Therapy

**DOI:** 10.1002/mds.27022

**Published:** 2017-06-08

**Authors:** Anders Christian Meidahl, Gerd Tinkhauser, Damian Marc Herz, Hayriye Cagnan, Jean Debarros, Peter Brown

**Affiliations:** ^1^ Medical Research Council Brain Network Dynamics Unit at the University of Oxford Oxford UK; ^2^ Nuffield Department of Clinical Neurosciences, John Radcliffe Hospital University of Oxford Oxford UK; ^3^ Department of Neurology Bern University Hospital and University of Bern Bern Switzerland; ^4^ Institute of Neurology University College London London UK

**Keywords:** deep brain stimulation, Parkinson's disease, brain–computer interface, essential tremor, closed‐loop

## Abstract

Continuous high‐frequency DBS is an established treatment for essential tremor and Parkinson's disease. Current developments focus on trying to widen the therapeutic window of DBS. Adaptive DBS (aDBS), where stimulation is dynamically controlled by feedback from biomarkers of pathological brain circuit activity, is one such development. Relevant biomarkers may be central, such as local field potential activity, or peripheral, such as inertial tremor data. Moreover, stimulation may be directed by the amplitude or the phase (timing) of the biomarker signal. In this review, we evaluate existing aDBS studies as proof‐of‐principle, discuss their limitations, most of which stem from their acute nature, and propose what is needed to take aDBS into a chronic setting. © 2017 The Authors. Movement Disorders published by Wiley Periodicals, Inc. on behalf of International Parkinson and Movement Disorder Society

Deep brain stimulation (DBS) has become an established and reversible treatment of motor symptoms in essential tremor, dystonia, and severe Parkinson's disease (PD), resulting in reductions in clinical impairment and increased quality of life in patients.^1^, ^2^, ^3^, ^4^, ^5^, ^6^, ^7^ The DBS systems used in current clinical practice effectively operate open‐loop so that stimulation parameters remain constant over time between visits to the clinician. With few exceptions, stimulation is applied at regular and high frequencies, in excess of 100 Hz. Such high frequencies of stimulation are believed to create an acute information lesion, whereby transmission of aberrant neural activity is attenuated.^8^, ^9^, ^10^ At the same time, however, any residual physiological communication may be impaired in the stimulated brain circuit.^11^ It is perhaps no surprise then that the application of DBS in movement disorder patients is limited by side effects such as speech impairment, psychiatric symptoms, and antagonistic worsening in some motor functions, which are all thought to arise from interactions between the stimulation and local brain circuits.^11^, ^12^, ^13^, ^14^, ^15^, ^16^ Such side effects could potentially be reduced by sparing neural circuits from high‐frequency stimulation when dysfunction is limited or by patterning stimulation so that it is far more selective for dysfunctional neural dynamics. In line with this, recent studies suggest that DBS might be more efficient and efficacious if modulated in response to the inferred state of activity in pathological brain circuits.^17^, ^18^

The primary goal of DBS that adapts to the current state of pathological activity, so called adaptive DBS (aDBS), is to increase the specificity of the intervention, and thereby widen the therapeutic window. A secondary goal is to reduce power drains on the implanted pulse generator (IPG). Improvements in battery technology have lessened but not negated the gains to be had from reduced power consumption. A significant proportion of patients are unsuitable for rechargeable IPG systems,^19^ and those that are would benefit from the ability to recharge less frequently. However, perhaps the most exciting impact of reduced power demands might be the possibility of reducing rechargeable battery size sufficiently to enable skull mounted IPGs.^20^, ^21^, ^22^

How might aDBS systems infer the current state of activity in the pathological circuits with which they interact? Essentially information can be derived from peripheral measures of motor state, such as the monitoring of tremor or involuntary writhing movements (dyskinesias), directly from recordings of brain activity, or through a combination of these approaches. Where deep brain signals are recorded these must be robust over many years, and for this reason local field potential activity is preferred over microelectrode recordings of neurons, even though features of both may correlate with symptoms.^23^, ^24^, ^25^, ^26^, ^27^ Microelectrode recordings are, however, less stable over time. Also critical is to consider the nature of those pathological circuits in which activity is inferred. These may be mechanistically or causally related to the symptoms or serve as faithful markers of the primary circuit dysfunction. In the former case, stimulation can be specifically patterned to maximally disrupt the underlying causal circuit dynamics, as for example, in the case of phase‐responsive forms of stimulation being pioneered for the treatment of tremor.^28^ In the latter case, the DBS intervention is necessarily more generic, and its delivery is controlled by the inferred state of the unmeasured or unknown primary circuit dysfunction. An example of a generic DBS approach is the stereotyped high‐frequency stimulation that forms conventional DBS (cDBS). This may be modulated according to the amplitude of signals that need not directly relate to the primary circuit dysfunction, but provide indirect evidence of the severity of the latter. We term this *amplitude‐responsive aDBS* and distinguish it from the more specific phase‐responsive DBS currently being developed for tremor treatment (Fig. 1). An example of amplitude‐responsive aDBS is DBS which is delivered according to the level of beta (13‐30 Hz) local field potential (LFP) activity recorded in the subthalamic nucleus (STN); we do not know for sure whether beta activity is causally important or not, but it correlates with motor impairment in PD patients undergoing functional neurosurgery^29^ and thereby provides a surrogate for whatever may be mechanistically at play.

**Figure 1:**
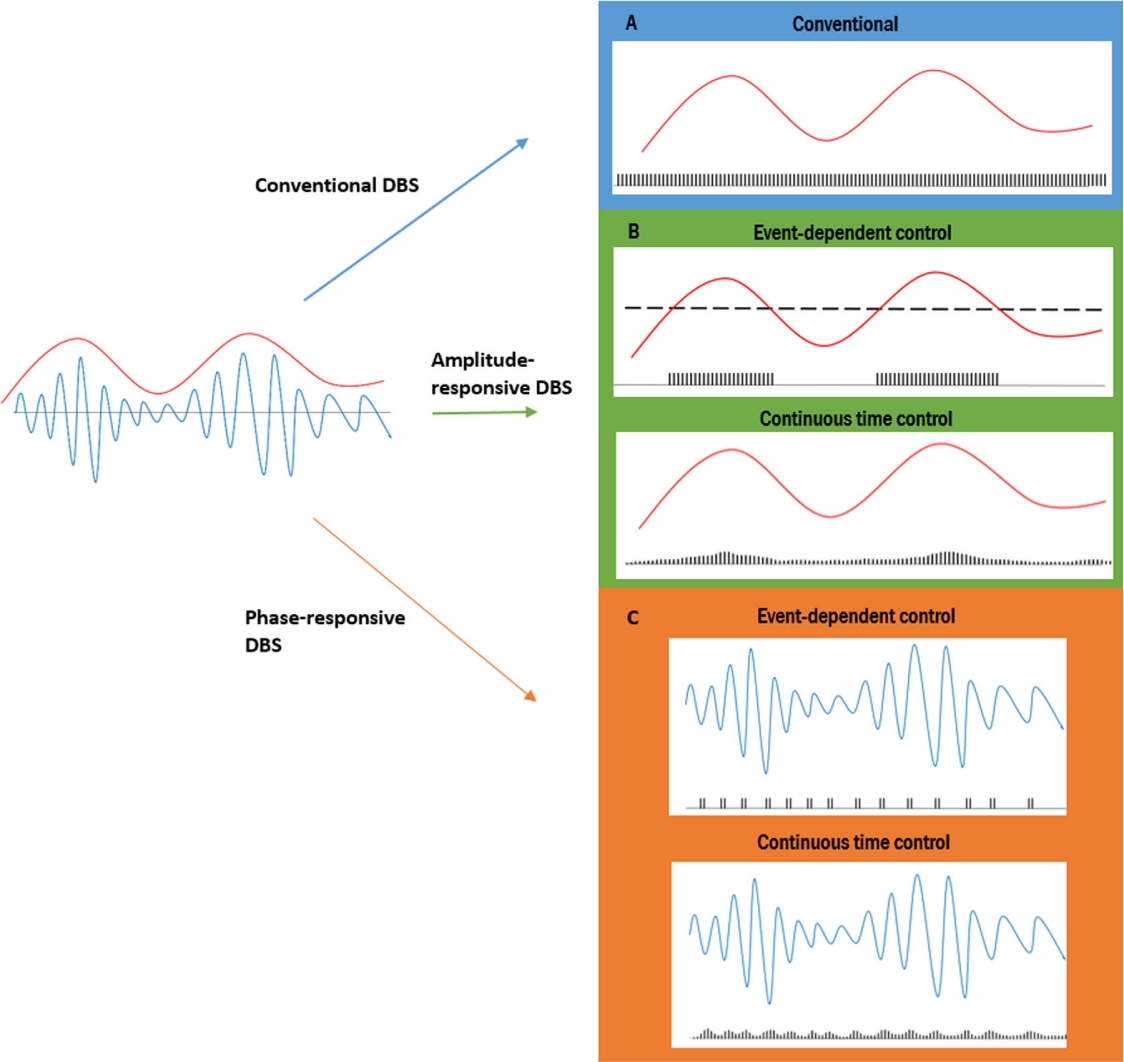
Schematic summary displaying different forms of DBS. A shows conventional DBS where pulses occur at a constant frequency. B depicts two forms of amplitude responsive DBS; upper green panel, event‐dependent control where stimulation is triggered and terminated when a signal, like beta‐amplitude, rises above and falls below a threshold, respectively and green lower panel, continuous‐time control where stimulation varies proportionately to the amplitude of the signal. C shows phase‐responsive DBS where pulses of high‐frequency stimulation are timed to a particular phase by either event‐dependent (upper orange panel) or continuous time control (lower orange panel). [Color figure can be viewed at wileyonlinelibrary.com]

The choice between amplitude and phase‐responsive approaches primarily depends on the maturity of our knowledge about underlying causal circuit dysfunction. In the case of tremor it is reasonable to attribute the peripheral disturbance to central oscillators that empirical evidence suggests can be controlled at the level of the motor cortex or thalamic cerebellar receiving areas.^30^, ^31^ For most conditions, however, we have no compelling evidence as yet that one or other circuit feature lies at the heart of the motor disturbance. For these it is best to use generic high‐frequency DBS and to control its delivery through the monitoring of surrogates such as the beta activity recorded in the STN LFP.

In this review, we first consider the evidence motivating further development of amplitude‐responsive and phase‐responsive aDBS, the limitations of trials performed to date, and what remains to be done if aDBS is to be translated in to clinical practice.

## Applications and Supporting Evidence

### Amplitude‐responsive aDBS

Amplitude‐responsive aDBS was systematically tested in parkinsonian humans for the first time in an acute trial in 2013^17^ after a landmark study of aDBS in nonhuman primates yielded promising results.^32^ Unilateral DBS was performed for about 10 minutes in 8 PD patients in the STN up to 7 days after electrode implantation. High‐frequency DBS was delivered whenever the beta activity exceeded a threshold amplitude of beta in the STN. This resulted in a 50% reduction in the contralateral upper limb Unified Parkinson's Disease Rating Scale (UPDRS) motor score assessed by blinded evaluation compared to no stimulation. When compared with cDBS, aDBS was significantly (27% absolute reduction) more effective and resulted in a 56% reduction in time on stimulation. An important element of the study was the comparison of aDBS to random, intermittent stimulation unlocked to LFP bursts of beta activity. Random DBS resulted in trivial UPDRS change in blinded assessments despite involving almost exactly the same time on stimulation as aDBS. Hence randomness of stimulation could not account for the clinical efficacy of aDBS.

Nevertheless, this proof of principle study was limited by short stimulation times and unilateral application that did not allow assessment of axial signs and gait. These limitations were addressed in a follow‐up study of bilateral aDBS in 4 PD patients tested over stimulation sessions lasting up to about 2 hours.^33^ Here aDBS improved blinded total UPDRS III scores by 43% when compared with no stimulation, similar to the improvement in cDBS studies in which blinded video assessments have been made.^34^, ^35^ aDBS also resulted in a 55% reduction in stimulation time, which could be decreased further by additional administration of levodopa. The bilateral sensing and stimulation and longer stimulation times in this study enabled the assessment of gait and axial signs, which presented an improvement on the earlier study. However, no direct comparisons between aDBS and cDBS were made.

An additional animal study trialed amplitude‐responsive DBS of the STN in a single nonhuman primate MPTP‐model of PD.^36^ The study found aDBS to be as good, if not better, than cDBS in reducing rigidity despite stimulation being present for only about 50% of the time and that reaching speed was not changed by either treatment. However, only cDBS reduced the speed of the return from a reach with the upper limb in a cue reaching task.

Together these studies suggest that, at least under acute testing conditions, aDBS is effective despite involving substantially less energy than cDBS. This reduction in energy raises the hope that side effects will be correspondingly less for a given voltage or current of stimulation than with cDBS and that the goal of a widened therapeutic window might be met. Several studies support this hypothesis. Both a case report and a case series of bilateral aDBS in freely moving PD patients have used a scalar approach where stimulation voltages varied according to LFP beta changes through a personalized algorithm, rather than the binary approach of turning DBS on and off used in other studies (Fig. 1B, lower green panel). aDBS substantially reduced the dyskinesias in patients on medication.^37^, ^38^ In addition, the acute impact of aDBS on speech was assessed in 10 PD patients in a further study.^28^ The patients received bilateral aDBS and cDBS, but only cDBS caused an acute deterioration in speech, assessed through the blinded assessment of the speech intelligibility test. This was despite the better motor improvement with aDBS in this cohort.

One concern with the use of beta activity as a feedback biomarker has been what might happen during movement, when beta activity is suppressed.^36^ In practice this has not been a problem in patient studies, as much of the motor UPDRS involves active movement, and yet this is improved during beta amplitude‐responsive aDBS. This might be because bradykinetic movements are associated with poor beta reactivity so that impaired movement may still be accompanied by beta activity that is sufficient to trigger stimulation.^39^, ^40^, ^41^, ^42^ Dopaminergic medication improves both beta reactivity and movement, but stimulation under these more physiological circumstances is less relevant.

Another issue has been how might amplitude‐responsive aDBS work in PD. This was addressed in a recent electrophysiological analysis of previously reported data sets.^43^ The study showed that aDBS selectively trims long duration beta bursts and increases the number of short beta bursts, relative to no stimulation and cDBS. Critically, the amplitude of beta bursts scales with their duration so that longer bursts signal greater pathological oscillatory synchronization within the local neural population. In line with this, the prevalence of short and long beta bursts negatively and positively correlated with motor impairment, respectively.

What about amplitude‐responsive DBS for tremor? Here there have been several trials. These, unlike the above, have been performed in chronically implanted patients, although they still remain acute in nature. The earliest study used not the severity of tremor itself to trigger stimulation onset, but surface electromyographic (EMG) activity over the deltoid muscle signalling upper limb activation. This was then used to trigger periods of thalamic DBS in five patients with severe intention tremor due to multiple sclerosis, 2 of whom had pronounced functional benefit.^44^ However, the absence of a control condition without stimulation meant that it was unclear whether benefit was due to lesion effects or adaptive DBS. Subsequent studies have used the severity of tremor recorded with peripheral inertial‐based sensors and/or surface EMG activity as the feedback signal, a personal computer to process the signals, extract key features and implement the control algorithm, and electromagnetic telemetric communication with the IPG. Control algorithms usually involve simple thresholding of processed signals, but can be more sophisticated, and utilize additional information about whether the patient is engaged in rest, posture, or action.^45^ The nature of the telemetric link and implanted IPG has meant that stimulation is then controlled with an approach of turning DBS on and off.

The clinical efficacy of aDBS for tremor can be difficult to ascertain from these more recent trials, as some only formally evaluate the success of tremor prediction^45^ or report changes in tremor amplitude, rather than changes in clinical rating scales more readily related to disability. Yamamoto and colleagues^46^ aimed to treat intention tremor in patients with severe essential tremor and triggered high‐frequency thalamic DBS whenever spectral power at tremor frequency in the EMG from biceps brachii exceeded a customized threshold. This led to complete resolution of intention and action tremor, as assessed unblinded using corresponding elements of the essential tremor rating scale in all 4 patients tested. The average time on stimulation was not reported, however. Another important trial explored amplitude‐responsive aDBS in tremor‐dominant PD.^47^ Five patients were studied and unilateral high‐frequency DBS was delivered whenever the tremor power detected by a Smart Watch (LG G‐watch) exceeded a threshold. Tremor power was reduced on average only by 37% relative to baseline, but this was achieved with voltages that were almost 80% lower than those used for cDBS. Stimulation was also delivered for only 50% of the time. However, the system had difficulty in distinguishing tremor from episodes of voluntary movements, and these were therefore excluded from analysis.

### Phase‐responsive aDBS

Where we have good reason to suspect that a particular neural activity directly relates to symptomatology we can potentially increase the efficiency and selectivity of electrical stimulation by patterning the latter so that it specifically modulates the causal neural activity. In a landmark study from 2011, Rosin and colleagues^32^ performed a form of phase‐responsive aDBS in the MTPT model of PD in 2 nonhuman primates. They showed aDBS to be more effective at attenuating motor symptoms compared to cDBS when applying brief, high‐frequency bursts of simulation to the globus pallidus interna, 80 ms after the detection of spikes in single neurons recorded in the ipsilateral M1. The 80‐ms delay was critical in improving motor impairment and corresponded to the cycle of the 9‐15 Hz beta band oscillations typical of this model. Recently, phase‐responsive aDBS has been applied to treat tremor.^48^ The assumption here is that oscillatory activity in brain circuits drives peripheral tremor oscillations with a more or less fixed time delay, so that delivering DBS pulses at particular phases of the cycle of the peripheral tremor will also mean that such stimulation is phase locked (albeit with a fixed offset) to the pathological neural activity. Dynamical systems theory and empirical data suggest that there should be phases at which impulses will increase or decrease the amplitude of the oscillations, just as there are points in the excursion of a child's swing at which a push will impede or increase the swing cycle.^49^ Phase‐responsive aDBS for tremor aims to selectively stimulate at those phases that attenuate tremor amplitude. Stimulation consists of bursts of high‐frequency pulses timed to a particular phase of the tremor and delivered to the ventrolateral thalamus (Fig. 1C, upper orange panel). Sustained locking of DBS to the optimal phase for amplitude suppression led to clinically significant tremor relief in 3 of the 5 patients with essential tremor tested, despite delivering less than half the energy of conventional high‐frequency stimulation.^48^

Nevertheless, phase‐responsive aDBS has not been delivered for longer than 30 seconds thus far, and longer trials are essential. Only then will it be possible to test whether this form of stimulation will give sustained tremor suppression with an improved side effect profile due to the lower energy delivery and selectivity for those oscillations that are phase locked to the stimulation. In the longer term it will also be interesting to see if this technique can improve akinesia and rigidity when stimulation is phase‐locked to beta activity in the STN.

With regard to parkinsonism, coordinated reset neuromodulation, where brief bursts of stimulation pulses are asynchronously delivered to different parts of the subthalamic region, has in proof‐of‐concept studies in primates and humans shown after effects with beta LFP suppression and UPDRS score improvements.^50^, ^51^ This is hypothesized to be a result of neuronal phase resetting followed by plastic changes in local neural circuits. These proof‐of‐concept studies indicate that DBS phase targeting in PD patients could have relatively sustained symptom‐attenuating effects.

In practice the successful application of phase‐responsive aDBS will also entail consideration of how best to keep pathological oscillators in the desynchronized state. This is because the amplitude attenuating effects of aDBS at suppressive phases build up over many cycles. Phase‐responsive aDBS alone would mean a delay in tremor suppression whenever significant tremor emerged. In the study by Cagnan and colleagues^48^ when phase‐responsive aDBS induced clinically significant tremor suppression it did so over the course of several seconds in some patients with essential tremor, but then the weak tremor precluded reliable identification of tremor phase and effectively led to random stimulation at low frequency. This tremor remained suppressed, raising the possibility that random low‐frequency stimulation is sufficient to keep central tremor oscillators desynchronized once they have been suppressed.

In summary, phase‐responsive aDBS may be best thought of as a means of suppressing tremor and controlling episodes of break‐through tremor, but a necessary adjunct may be intercurrent stimulation to maintain tremor in its attenuated state. In this regard, phase‐responsive aDBS might be considered similar to conventional high‐frequency DBS in that the acute effect of both on tremor is delayed, and the clinical benefits accrue because once tremor is suppressed it is maintained in this state by continued stimulation. The major difference is that aDBS may involve lower overall frequencies of stimulation and less energy delivery.

## Limitations and Roadblocks

Research on aDBS to date raises several issues.

### Leveraging Existing Electrode Designs

Currently, aDBS is delivered through standard DBS electrodes. Until recently, these were exclusively quadripolar, which leads to constraints when brain signals are recorded through the same electrode and used for feedback control. This is because the difference in voltage between stimulation pulses and pathological LFP activity is so great that common mode rejection is necessary to recover spontaneous LFP activity. Stimulation therefore has to be delivered at either of the 2 middle contacts of the quadripolar electrodes, and the LFP recorded bipolarly from the neighboring pair of contacts. The net result is that fewer electrode contacts are available for aDBS performed in this way than for cDBS. This, however, may be less of a problem as the number of electrode contacts increases as electrode design improves. Octopolar directional and nondirectional electrodes are already available and thin‐film probe technology has made realistic the possibility of high density, high resolution, multicontact DBS electrodes.^52^

### Difficulty in Assessing Efficacy

Trials of aDBS in which feedback is derived from LFP activity have necessarily been carried out 2 to 7 days after electrode implantation because of the need to record directly from implanted electrodes. This is problematic. First and foremost, patients may experience substantial symptom relief as a result of the so‐called “stun effect” resulting from local trauma,^53^ and it is for this reason that most centers delay postoperative programming.^54^ The exact duration of the stun effect is not fully established, but recent studies have found UPDRS score improvements off stimulation postoperatively compared to the preoperative state for up to 6 months after electrode placement.^53^ It has also been shown that changes in both impedance and LFPs occur after electrode implantation, particularly within the first 24 hours.^5^, ^55^, ^56^ For these reasons, acute perioperative aDBS findings may not necessarily be representative of the chronic state.

Another disadvantage of studying the effects of aDBS in the acute postoperative setting is the limited time available and confounding postoperative fatigue leading to drifts in baseline performance. Time constraints and stun effects limit the extent to which cDBS may be optimized, and this may explain why cDBS fails to have an appreciable therapeutic effect in some studies.^28^, ^37^ This complicates the interpretation of the acute perioperative contrast of the performance of aDBS and cDBS, as neither may be representative of that following recurrent optimization in the chronically treated patient. Some of these limitations could be circumvented by contrasting aDBS and cDBS acutely at the time of battery change. Here stun effects are absent and the experimenter benefits from the knowledge of the clinically optimized stimulation parameters.

### Difficulty in Assessing Side Effects

Although there have been some encouraging initial reports of reduced side effects during aDBS, those side effects seen and tested in the acute postimplantation period may also be unrepresentative of the chronic state. Dyskinesias may, for example, be problematic when stimulation is combined with dopaminergic medication following surgery, but this will be less so once the stun effect abates and once medication has been titrated over time.^37^ The speech impairment that was shown with acute cDBS, and was absent with aDBS, might also disappear with optimisation of cDBS stimulation site and parameters over time.^28^ In reality, side effects (and efficacy) should be assessed during chronic treatment in the patient's everyday environment, with the clinical team afforded the opportunity to optimize both DBS therapy and medication.

### Insufficient Time for Adaptive Effects

The mechanisms by which DBS exerts its therapeutic effects are still not fully understood and are very likely multimodal involving various time‐dependent mechanisms such as acute local neuromodulation, changes in synaptic plasticity, and long‐term anatomical reorganization.^10^ Any potential, adaptive, long‐term effects cannot be studied in the acute setting and will need to be assessed during chronic treatment. Interestingly, a tendency for the time on stimulation to progressively fall during aDBS has been reported, suggestive of short‐term plasticity.^17^ Such plasticity has also been noted in computational studies of desynchronizing brain stimulation, including DBS,^57^ and may contribute to the carry‐over effects of high‐frequency electrical stimulation triggered by automated seizure detection noted in patients with epilepsy.^58^

The converse of the above is maladaptive plasticity, and this is perhaps commonest in the treatment of essential tremor by conventional thalamic DBS. Here it leads to habituation to the effects of DBS over time and gait ataxia,^59^, ^60^ and prolonged trials are necessary to determine whether this also occurs with aDBS.

### Use of Simplistic Feedback Signals and Control Algorithms

Hitherto, feedback signals have generally been one dimensional, such as beta power or tremor severity. However, this may be suboptimal. For example, beta power in the STN correlates with bradykinesia and rigidity, but it does not do so with tremor.^18^, ^23^ Thus aDBS systems that rely on beta activity feedback run the risk of not controlling tremor. So far, in practice, this has not been the case, perhaps because tremor is one of the features that is most susceptible to the stun effect or because the stimulation intensity necessary for tremor suppression was below that needed for beta suppression, and bursts of aDBS were given sufficiently frequently for tremor to remain suppressed. Nevertheless, there is a case to be made for combining both beta activity and inertial sensor activity in a dual feedback control loop (Fig. 3). Indeed, there are many other possible brain and peripheral signals that correlate with motor impairment that could, in principle, be combined for more precise control of different symptoms.^18^, ^61^, ^62^, ^63^

**Figure 2:**
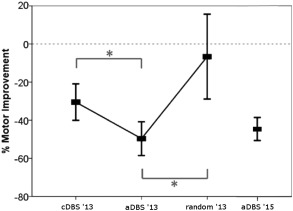
Improvements in motor scores assessed blinded in the studies of Little et al. 2013 and Little et al 2015. Data are presented as the mean ± standard error of the mean percentage change in UPDRS scores.

**Figure 3:**
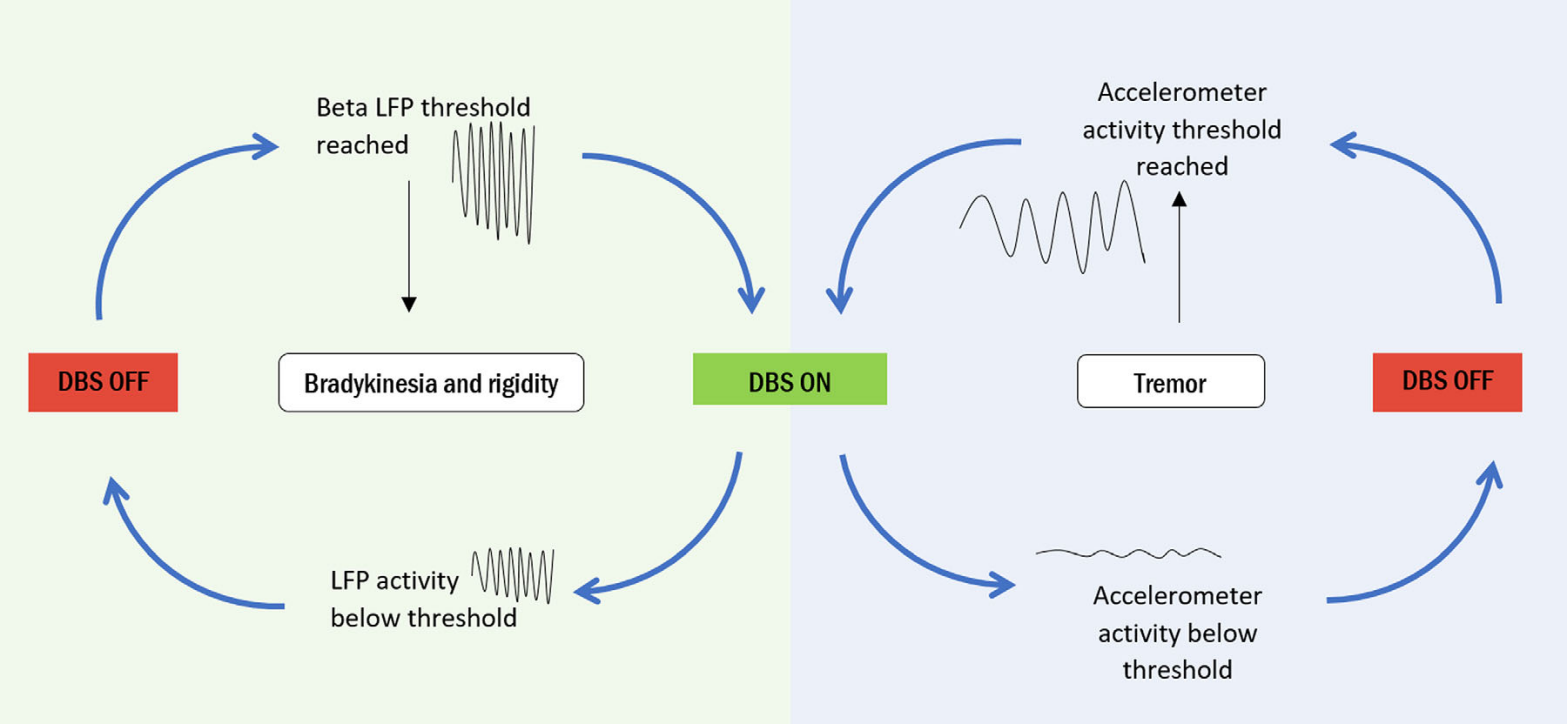
Potential Combined Beta‐Tremor Control loops. DBS stimulation is triggered whenever one or other or both signals cross independent thresholds and is terminated whenever both signals have fallen below their thresholds. [Color figure can be viewed at wileyonlinelibrary.com]

However, the potential use of multiple feedback signals raises issues of redundancy, sufficiency, and tractability. Is the information from additional signals redundant? Are the benefits to be had with existing biomarkers, if borne out with chronic aDBS, sufficient in offering a step improvement in therapy? Are other candidate feedback signals tractable and practical in combination or alone? Consider, for example, the recording of the STN high‐frequency oscillations over 200 to 400 Hz and the modulation of these by the amplitude of local beta activity (termed *phase‐amplitude coupling*), which have been demonstrated to correlate with bradykinesia and rigidity.^64^, ^65^ Detecting the very low amplitude of STN high‐frequency oscillations in the setting of high‐frequency stimulation delivered through the same electrode is likely to be very difficult and power demanding. An alternative might be to record phase‐amplitude coupling at the level of the motor cortex,^66^ but it is unclear whether cortical high‐frequency oscillations are any different in amplitude to those in the STN. On the other hand, recent work suggests that phase‐amplitude coupling may be at least partly driven by asymmetries in the waveform of beta band oscillations,^67^ and in this case there would certainly be advantages to cortical recordings as cortical beta is significantly greater in magnitude than STN beta due to the improved spatio‐temporal integration afforded by cortical lamination.

In addition to the simplicity of current feedback signals, amplitude‐responsive aDBS has most often relied on an on‐off control policy. This is an “event‐dependent control,” whereby the change of state (on/off) of the stimulation depends on beta amplitude crossing a predefined threshold (Fig. 1B, upper green panel). Although this can work well, for example, in the thermostatic control of central heating, it ignores information that might be afforded by the continuous evolution of the feedback signal. For example, is room temperature dropping fast, in which case there might be an advantage in turning on the central heating earlier or higher. Continuous evolution of the signal is considered in continuous‐time control strategies (Fig. 1C, lower green panel). So far the latter have been implemented in the simple form of the proportional control used by Rosa and colleagues.^37^ Here high‐frequency stimulation was controlled so that its intensity depended proportionally (with a constant gain) on the amplitude of beta activity at all times. An alternative approach would be to implement proportional‐integral‐derivative control, a well‐established and successful engineering method,^68^ recently successfully applied in automated insulin delivery systems.^69^ This form of control responds to and anticipates changes in the feedback signal both rapidly and efficiently. Moreover, continuous‐time control lends itself to implement more advanced control approaches such as nonlinear stochastic dynamic control or multivariable control. A simple example of the latter would be a dual‐control system, whereby central beta activity and inertial signals related to peripheral tremor are simultaneously controlled by stimulation, or different spectral elements of the local field potential activity are simultaneously controlled but with different set references. This very versatility of continuous‐time control, however, again underscores the need to consider issues of redundancy, sufficiency and tractability in aDBS design. Furthermore, it is critical that such design considers biological constraints. One specific constraint with respect to DBS is that stimulation effects have a threshold so that continuous‐time control below this threshold may be relatively superfluous and inefficient. Accordingly, future implementations of aDBS may involve hybrid control whereby continuous‐time control approaches kick in once a threshold signal value is surpassed.

Finally, when considering multidimensional feedback and more sophisticated control systems it is important to bear in mind limitations on the embedded computing power possible in implanted systems and the battery power burden that these operations may bring. Amplitude‐responsive aDBS has been estimated to decrease total electrical energy delivered by about 130 µW per side,^17^ whereas the energy consumed by a low‐energy circuit underpinning a single‐channel power classifier should be no more than about 10 µW. Thus unidimensional signals and a simple control policy can achieve very significant energy savings, but this remains to be seen if more complex approaches are sought.^70^

### Lack of Optimization

Hitherto the threshold of on‐off control or the gain of the proportional control have been determined heuristically. This means that although beneficial, they may not still be optimal. Indeed, there are multiple parameters to select and optimize in DBS, including contacts to be stimulated, pulse width, stimulation frequency, and stimulation intensity. In amplitude‐responsive DBS additional variables are the frequency‐band of interest and smoothing of the feedback signal, the threshold, the steepness of any ramp in stimulation introduced to limit side effects such as paraesthesia, and the duration of any lock‐out period before stimulation can be triggered again. In phase‐responsive DBS additional variables are the selection of the phase for stimulation, and the number and frequency of pulses to be delivered at that phase. So far choices have been made on empirical grounds, but the very presence of an informative feedback signal offers the possibility of automatic, on‐line, comprehensive optimization of variables to achieve maximal effect on the feedback signal—something that is just not feasible to achieve through clinical assessments.

### Need for Patient Selection

With only acute trials performed thus far it is difficult to predict which patients might respond best to chronic aDBS. Patient selection is likely to be particularly important when applying more specific phase‐responsive techniques. Indeed, in the study by Cagnan and colleagues,^48^ only patients with essential rather than dystonic tremor had the potential for marked tremor suppression, and even within those with essential tremor there were factors such as tight tuning of tremor and the presence of a single dominant limb oscillation that favored a good response. Broadening phase‐responsive stimulation to those with more than 1 pattern of limb oscillation over time may require systems that can track the principal tremor axes characterizing independent oscillators and optimize the phase of stimulation accordingly.

### Questions About Long‐term Stability

What if the feedback features and the thresholds or gearings used in aDBS change over time, with medication changes, alteration in the brain–electrode interface, or disease progression? At present this question is totally unexplored, as aDBS has only been acutely trialed. However, for aDBS to be clinically useful it must be robust to changes over time and may need the functionality to perform auto‐identification and auto‐tuning so that the system can identify online and in real‐time optimal control parameters. As a minimum, it may be necessary to include a system that monitors the stability of the control loop. Such a system could, for instance, be implemented in a simple way by using a performance index such as the Integral Time‐weighted Absolute error, which can be performed with very little computing power.^68^ This would enable a fail‐safe approach whereby should the performance index exceed a given value, aDBS could be reverted to cDBS; then when the performance index returns to a suitable value, the aDBS algorithm would be reactivated.

## Conclusion: The Implantable Technology Gap

Adaptive DBS is a promising therapeutic development that may, in time, complement other innovative approaches to deep brain stimulation, such as novel pulse parameters or current steering.^63^ The hope, too, is that it might eventually be rolled out to movement disorders other than parkinsonism and tremor, as knowledge of correlating biomarkers or causal neural patterns matures. Phase‐responsive aDBS might also lend itself to the control of some of the intractable childhood epilepsy syndromes characterized by long and rhythmic sequences of epileptic activity. However, the aDBS studies performed to date have significant limitations, and these stem from the fact that the investigations have all necessarily been acute, either because the only way to reliably record low‐amplitude subcortical LFPs and to rapidly react to changes is to externalize electrode connections in the short‐lived perioperative window or because electromagnetic telemetry links to chronically implanted IPGs are slow, sensitive to alignment, and unsuitable for chronic trials in real‐life situations. We are therefore fast approaching an impasse in which the utility and chronic viability of aDBS cannot be further explored without suitably functioning, implantable, medical grade devices. Overcoming this roadblock will require the continued development of permissive technology by the medical devices industry.

## Author Roles

1) Research project: A. Conception, B. Organization, C. Execution; 2) Statistical Analysis: A. Design, B. Execution, C. Review and Critique; 3) Manuscript: A. Writing of the first draft, B. Review and Critique.

A.C.M.: 1A, 1B, 1C, 2A

G.T.: 2B, 2C

D.M.H.: 2B, 2C

H.C.: 2B, 2C

J.D.: 2B, 2C

P.B.: 1A, 1B, 1C, 2B, 2C

## Full financial disclosures for the previous 12 months

P.B. is supported by the Medical Research Council (MC_UU_12024/1) and the National Institute for Health Research Oxford Biomedical Research Centre and A.Ms DPhil‐studies are supported by the Rosetrees Trust. GT is supported by a research fellowship by the European Academy of Neurology (EAN). DMH is supported by European Commission (MSCA individual fellowship). HC is supported by an MRC fellowship.
